# Comparison of the different methods of width estimation in unerupted canine and premolars

**DOI:** 10.1186/s12903-024-04053-8

**Published:** 2024-04-20

**Authors:** Tania Ghasemi, Morteza Sabbaghzadeh, Melika Mollaei, Maysam Mirzaei

**Affiliations:** 1 Department of Orthodontics, Dental Research Center, Faculty of Dentistry, Mazandaran University of Medical Sciences, Sari, Iran; 2 Babol University of Medical Sciences, Babol, Iran; 3 Student Research Committee, Dental Research Center, Faculty of Dentistry, Mazandaran University of Medical Sciences, Sari, Iran; 4 Oral Health Research Center, Health Research Institute, Babol University of Medical Sciences, Babol, Iran

**Keywords:** Mixed dentition, Space analysis, Tanaka Johnston, Moyers, Linear regression

## Abstract

**Background:**

There are different methods for determining the required space for unerupted teeth. However, the accuracy of these techniques varies depending on ethnic differences. Therefore, the current study was performed to compare the accuracy of four methods for estimating the mesiodistal width of unerupted canines and premolars in a population of northern Iran.

**Methods:**

The present cross-sectional study was conducted on 50 pairs of dental casts of patients aged 12–24 years old. The mesiodistal width of the teeth was measured with a digital caliper by two observers (ICC < 0.9), and the mean value was recorded. The space required for eruption of canines and premolars was obtained by the Tanaka-Johnson formula and the Moyers tables and compared with the actual value by paired t test.

**Results:**

The Tanaka-Johnson formula had overestimation in the maxilla and mandible, which was statistically significant (*p* < 0.001). The values obtained from the Moyers tables in different confidence levels were not accurate. However, the 65% level for the mandible had almost no difference from the actual value (*P* = 0.996 and r^2^ = 0.503). Furthermore, linear regression was obtained based on the total mesiodistal width of the maxillary first molar and mandibular central incisor (maxilla: Y_x_= 0.613X + 2.23 and mandible: Y_m_= 0.618X + 1.6) and the total mesiodistal width of the mandibular first molar and maxillary central incisor in each jaw (maxilla: Y_x_ = 0.424X + 5.021 and mandible: Y_m_ = 0.447X + 3.631).

**Conclusion:**

The Tanaka-Johnson method was overestimated in the population of northern Iran. The 85% and 75% confidence levels of the Moyers table have the best clinical results for the maxilla and mandible, respectively. Regression based on maxillary first molars and mandibular central incisors has better results.

## Introduction

Impacted maxillary canines are those that are encased in the alveolus and cannot erupt at the proper time in the dental arch. Maxillary canine impaction is reported to have a frequency of 2% which varies by ethnicity [1]. Caucasian populations are likely to develop palatal impaction, whereas buccal impactions are more frequent in Asians. Studies on orthodontic patients show that this condition is more prevalent in women [2].

Early diagnosis improves the prognosis of these canines allowing them to reach their proper position. Delayed treatments might result in root resorption of adjacent incisors in 48% of patients, necessitating future treatments such as orthodontic alignment, surgical treatments, or extractions [3]. Currently, multiple methods have been suggested to predict unerupted canines and premolars’ mesiodistal width including radiographic modalities, non-radiographic modalities, and the combination of both of them. Predicting tables and regressions are some of the non-radiographic methods used in estimating the width of unerupted teeth [4]; however, it is clear that none of these methods can be accurate. Furthermore, tooth size varies continuously among people and factors such as ethnicity and sex influence its variability. Therefore, the current techniques cannot be used for every population [5, 6].

Methods requiring radiography consume more time and costs for the patients and result in deformities especially around canines due to the different positions of teeth in the dental arch. Moreover, several graphs are essential for more accurate calculation [7]. The Tanaka-Johnston formula has advantages such as simplicity, non-invasive nature, and application in both arches and genders [8]; nevertheless, some studies claim that this method might overestimate/ underestimate the actual size of the teeth [9, 10]. Many studies have been conducted to evaluate the mesiodistal width of canine and premolar teeth based on the size of the erupted teeth and linear regression. A non-radiographic estimation method should be accurate, simple, clinical and specific to the same population [11]. Each of these investigations used a specific tooth for estimation, the most common being mandibular incisors [12, 13].

It has been claimed that different ethnicities show differences in the mesiodistal width of permanent teeth [14]. Therefore, the methods obtained by statistical studies in one society are not accurate enough for others [7]. On the other hand, the authors could not find similar studies conducted on the northern Iranian population specifically. Therefore, in the current study four methods of estimating the mesiodistal width of canines and unerupted premolars were compared to suggest the most suitable one for estimating the mesiodistal width of canines and premolars in a northern population in Iran. The null hypothesis was that the linear regression can offer the most similar findings to the actual size of the unerupted teeth.

## Materials & methods

The current cross-sectional investigation was carried out on 50 dental casts of patients between the ages of 12–24, referring to the dental clinic of Babol University of Medical Sciences, Babol, Iran. Convenience sampling was used to select the study population. Based on Geramy et al’s study [15], the sample size was estimated as follows:

$${\rm N }= \frac{{\left({\text{z}}_{1}-\raisebox{1ex}{$\propto $}\!\left/ \!\raisebox{-1ex}{$2$}\right.\right)}^{2}\text{*}{\text{S}\text{d}}^{2}}{{\text{d}}^{2}} \propto = 0.05, {\rm Sd}=0.35, {\rm d}=0.1^{(2)}$$

$${\rm N } = \frac{{\left(1.96\right)}^{2}\text{*}{\left(0.35\right)}^{2}}{{\left(0.1\right)}^{2}} \approx 50$$

An informed consent was obtained from all participants or their legal guardians if necessary. The patients had fully erupted teeth with mild or no crowding or spacing and had no previous orthodontic treatment. Patients with extra or missing teeth, veneers, fractures, caries, proximal restorations, and teeth anomalies were excluded. The casts were intact and free of nodules or bubbles.

**Fig. 1:**
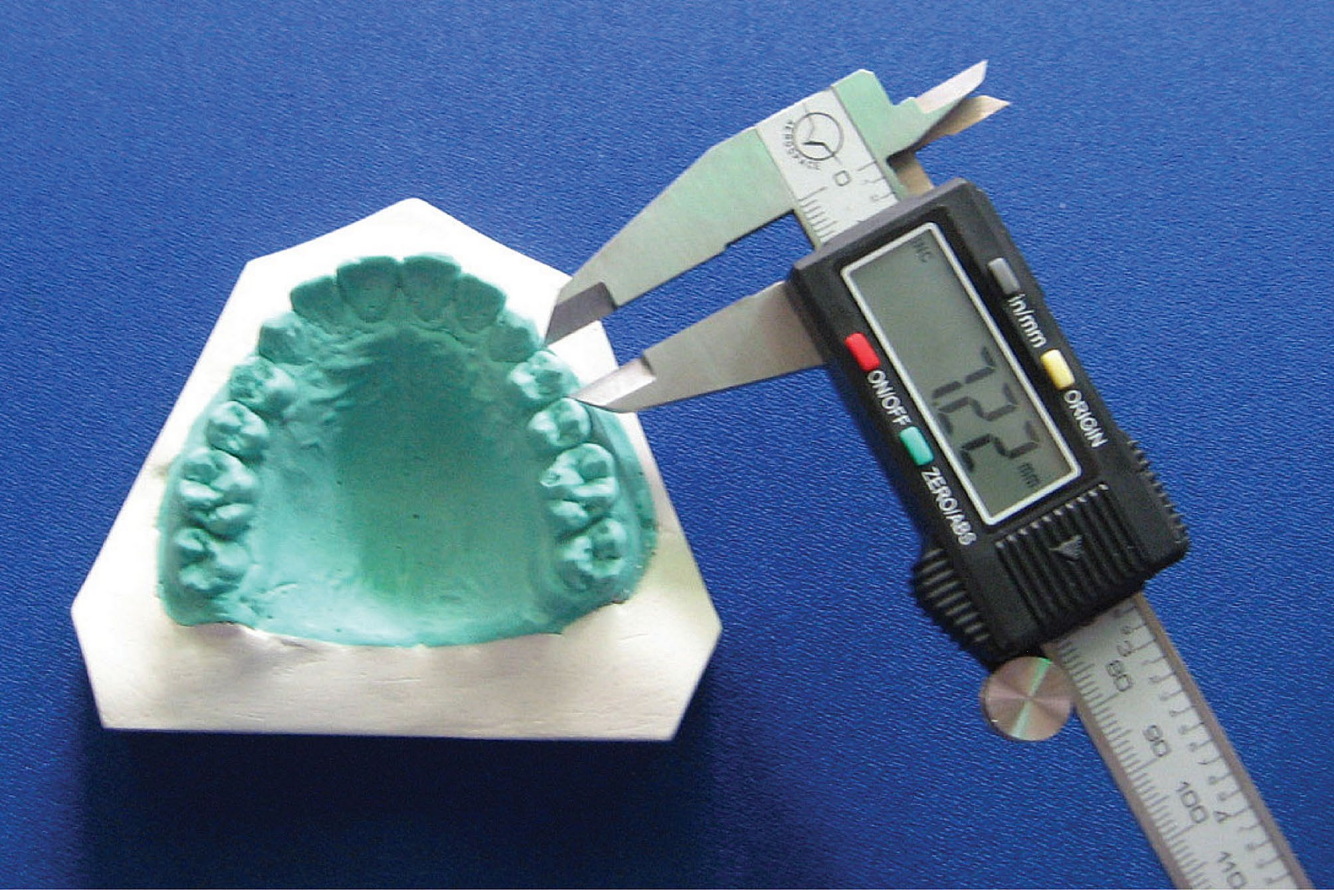
Measuring the mesiodistal width of teeth using calipers

The maximum distance between the contacts on the height of contour of the proximal walls parallel to the occlusal surface of the tooth with normal alignment was defined as the mesiodistal width. The mesiodistal width of each tooth was measured using a digital sliding caliper (INSIZE 1111, China) with a 0.01 mm accuracy (Fig. 1).

These measurements were performed by two studies separately. Intraclass correlation (ICC) was calculated to obtain agreement between them. The average values measured by both observers were used to perform all the analyses.

The average total mesiodistal width of the canine, and first and second premolars on both sides was estimated using four different methods as follows:

Tanaka-Johnston formula: The findings were then compared with the actual values using the paired t test.

(I) Mandible: Y_m_= ½ X + 10.5.
(II) Maxilla: Y_x_= ½ X + 11.

*Y*_*m*_: *Total mesiodistal width of mandibular canine and first and second and premolars*.

*Y*_*x*_: *total mesiodistal width of maxillary canine and first and second and premolars*

*X*: *total mesiodistal width of mandibular incisors*

(I) Moyers tables: The mesiodistal width of canine and premolars were estimated by these tables. However, since the total MW of the mandibular incisors is usually between the values determined in the table, the following formula was used to measure the exact value between the intervals:

$${\rm Y}= {\rm Y}_{1+} \frac{X-{X}_{1}}{{X}_{2} - {X}_{1}} * ({Y}_{2}-Y_{1})$$

*Y*: *Total mesiodistal width of canine and first and second premolars*.

*X*: *total mesiodistal width of mandibular incisors*

*X*_*2*_: the upper limit of the total mesiodistal width span of the mandibular incisors

*X*_*1*_: *The lower limit of the total mesiodistal width of the mandibular incisors*.

*Y*_*2*_: *The upper limit of the total mesiodistal width of canine and first and second premolars*.

*Y*_*1*_: *The lower limit of the total mesiodistal width of canine and first and second premolars*.

This method was used for all confidence levels of the Moyers table and the paired t test was used to compare the obtained values to the actual values.

(II) Linear regression: new equations were created based on the relationship between the total mesiodistal width of the first and second premolars and canine teeth with the total width of the maxillary first molars and the mandibular incisors, as well as the mesiodistal width of the canine and premolar teeth with the total mesiodistal width of the mandibular first molar and the maxillary incisors on both sides.

Data were entered into SPSS software version 18. The median, standard deviation, and minimum and maximum values were used separately for the maxilla and mandible on both left and right sides for actual measurements of the mesiodistal width of the teeth. The actual sizes of the teeth on both sides were compared by paired t tests. The average of the total mesiodistal width of the canine and premolars on both sides was used in all analyses. A significance level of less than 0.05 was taken into account.

## Results

In the current investigation 50 dental casts were examined to compare the accuracy of four methods of estimating the mesiodistal width of unerupted canines and premolars. The study population consisted of 17 (34%) men and 33 (66%) women.

For more accuracy, the MW of the first molars from one side to the other was measured by two observers. Thus, the internal agreement coefficient (ICC) was calculated in 24 teeth and reported to be greater than 0.9, which is at an appropriate level. The mean total width of the canine and first and second premolars on both sides of the maxilla and mandible was 21.79 ± 1.14 (ranging from 18.89 to 24.25) and 21.33 ± 1.15 (ranging from 18.87 to 24.30), respectively. The actual measurements are shown in Table 1.

The mesiodistal width of the left and right quadrant in each jaw was evaluated separately using the paired samples t test, and no significant difference was noticed (*P* < 0.05). Therefore, the average of canines and premolars on the left and right sides were used.

**Table 1:** Examining the maxillary and mandibular teeth’s actual mean sizes

Quadrant	Teeth	Maxilla			Mandible		
		Mean ± SD	Min	Max	Mean ± SD	Min	Max
**Right**	Central	8.81 ± 0.45	7.46	9.74	5.57 ± 0.31	4.85	6.26
	Lateral	7.04 ± 0.47	6.15	8.14	6.05 ± 0.34	5.41	6.74
	Canine	7.92 ± 0.42	7.11	8.95	6.89 ± 0.42	6.21	7.96
	First premolar	7.11 ± 0.39	6.09	8.16	7.18 ± 0.45	6.26	8.52
	Second premolar	6.81 ± 0.43	5.89	7.72	7.23 ± 0.45	6.28	8.81
	First molar	10.40 ± 0.47	9.36	11.51	10.97 ± 0.53	9.97	12.42
**Left**	Central	8.79 ± 0.45	7.66	9.63	5.59 ± 0.29	4.85	6.29
	Lateral	6.95 ± 0.50	5.96	8.25	6.09 ± 0.37	5.34	6.90
	Canine	7.87 ± 0.45	6.90	9.08	6.92 ± 0.46	6.02	8.11
	First premolar	7.06 ± 0.46	5.93	8.01	7.23 ± 0.44	6.22	8.51
	Second premolar	6.82 ± 0.43	5.47	7.78	7.21 ± 0.41	6.26	8.26
	First molar	10.36 ± 0.42	8.72	11.22	10.99 ± 0.50	9.84	12.06

Table 2 illustrates the comparison between the actual value and the value obtained by the Tanaka-Johnson formula for the mesiodistal width of the canine and first and second premolars. The paired samples t test revealed that the Tanaka-Johnson formula overestimated on average of 0.86 and 0.82 in the maxilla and mandible, respectively, which was statistically significant (*P* < 0.001).

**Table 2:** Comparison the exact measure of the total mesiodistal width of the canine and first and second premolars with the value obtained by the Tanaka-Johnson formula

Jaw	Teeth	Mean ± SD	R	R^2^	Difference of the means	*P* value*
**Maxilla**	Exact measure	21.79 ± 1.12	0.677	0.458	0.86	< 0.001
	Tanaka-Johnson	22.65 ± 0.61				
**Mandible**	Exact measure	21.33 ± 1.14	0.660	0.436	0.82	< 0.001
	Tanaka-Johnson	22.15 ± 0.61				

*p-value was obtained by paired t-test

As demonstrated in Table 3, the values obtained from the Moyers table with a confidence level of 75%, had an average underestimation of 0.09 compared with the actual size (*r* = 0.728) in the maxilla, which was not statistically significant (*p* > 0.05). Furthermore, the confidence level of 85% had an average overestimation of 0.24 compared with the real size (*r* = 0.729), which was statistically significant (*p* < 0.05), but not clinically significant. Moreover, findings in the mandible revealed that the 65% confidence level was not statistically significant (*p* > 0.05), and the 75% confidence level had an average overestimation of 0.34 (*r* = 0.707), which was statistically (but not clinically) significant (*p* < 0.05).

**Table 3:** Comparison of the exact measure of the total mesiodistal width of the canine and first and second premolars with the value obtained by the Moyers table

Jaw	Moyers confidence level (%)	Mean ± SD	Difference of the means	R	R^2^	*P* value*
**Maxillary canine and first and second premolars**	95	22.63 ± 0.57	0.85	0.737	0.543	< 0.001
	85	22.03 ± 0.61	0.24	0.729	0.531	0.040
	75	21.69 ± 0.63	-0.09	0.728	0.530	0.412
	65	21.40 ± 0.64	-0.39	0.720	0.518	0.001
	50	21.03 ± 0.68	-0.75	0.712	0.507	< 0.001
	35	20.67 ± 0.73	-1.11	0.701	0.491	< 0.001
	25	20.37 ± 0.72	-1.42	0.701	0.491	< 0.001
	15	20.02 ± 0.75	-1.77	0.695	0.483	< 0.001
	5	19.42 ± 0.79	-2.37	0.687	0.472	< 0.001
	Exact	21.79 ± 1.14	-	-	-	-
**Mandibular canine and first and second premolars**	95	22.81 ± 0.70	1.48	0.705	0.497	< 0.001
	85	22.09 ± 0.69	0.76	0.707	0.500	< 0.001
	75	21.68 ± 0.69	0.34	0.701	0.491	0.005
	65	21.33 ± 0.70	0.00	0.709	0.503	0.996
	50	20.87 ± 0.69	-0.46	0.713	0.508	< 0.001
	35	20.41 ± 0.70	-0.93	0.706	0.498	< 0.001
	25	20.07 ± 0.69	-1.26	0.713	0.508	< 0.001
	15	19.63 ± 0.68	-1.70	0.708	0.501	< 0.001
	5	18.91 ± 0.69	-2.42	0.710	0.504	< 0.001
	Exact	21.33 ± 1.15	-	-	-	-

*p-value was obtained by paired t-test

Linear regression was obtained based on the relationship between the total mesiodistal width of the canine and first and second premolars and the total mesiodistal width of the maxillary first molar and mandibular central incisor in each jaw (Table 4). The regressions were statistically significant:

*Maxilla*: *Y*_*x*_= *0.613 X + 2.23*

*Mandible*: *Y*_*m*_= *0.618X + 1.6*

*X: Total mesiodistal width of the maxillary first molar and mandibular central incisor on both sides*.

**Table 4:** Linear regression based on the relationship between the sum of the mesiodistal width of the canine and first and second premolars with the sum of the mesiodistal width of the maxillary first molar and mandibular central incisor

model		β	SE	t	*P* value	R	R^2^	SEE
**Y**_**x**_	X2	0.613	0.096	6.406	< 0.001	0.679	0.461	0.849
	Fixed value	2.23	3.054	0.732	0.468			
**Y**_**m**_	X2	0.618	0.096	6.408	< 0.001	0.679	0.461	0.857
	Fixed value	1.600	3.082	0.519	0.606			

* Linear regression analysis

*Y*_*x*_: *Total mesiodistal width of maxillary canine and first and second premolars (average of both sides)*

*Y*_*m*_: *Total mesiodistal width of the mandibular canine and first and second premolars (average of both sides)*

*X*_*2*_: *total mesiodistal width of the maxillary first molar and mandibular central incisor*

According to Table 5, another statistically significant regression was found based on the relationship between the total mesiodistal width of the canine and first and second premolars with the total mesiodistal width of the mandibular first molar and maxillary central incisor in each jaw, which is as follows:

*Maxilla*: *Y*_*x*_= *0.423 X + 5.031*

*Mandible*: *Y*_*m*_= *0.447 X + 3.631*

X: total mesiodistal width of mandibular first molar and maxillary central incisor on both sides

**Table 5:** Linear regression based on the relationship between the sum of the mesiodistal width of the canine and first and second premolars with the sum of the mesiodistal width of the mandibular first molar and maxillary central incisor

model		β	SE	t	*P* value*	R	R^2^	SEE
**Y**_**x**_	X3	0.423	0.075	5.627	< 0.001	0.630	0.397	0.898
	Fixed value	5.031	2.980	1.688	0.468			
**Y**_**m**_	X3	0.447	0.073	6.087	< 0.001	0.660	0.424	0.877
	Fixed value	3.631	2.911	1.247	0.218			

* *Linear regression analysis*

*Y*_*x*_: *Total mesiodistal width of maxillary canine and first and second premolars (average of both sides)*.

*Y*_*m*_: *Total mesiodistal width of the mandibular canine and first and second premolars (average of both sides)*.

*X*_3_: *total mesiodistal width of the mandibular first molar and maxillary central incisor*

Linear regression based on the sum of the mesiodistal width of the maxillary first molar and mandibular central incisor, had a higher correlation coefficient (r) and coefficient of determination (r^2^) and a lower standard error of estimation (SEE) compared to the mandibular first molar and maxillary central incisor regression.

## Discussion

This investigation aimed to compare the accuracy of four methods for estimating the mesiodistal width of unerupted canines and premolars in a northern population of Iran. The measurements were carried out by two observers for more accuracy. The findings suggested no significant difference between the mesiodistal width of the right and left teeth in both maxilla and mandible, which is consistent with the findings of some other studies [11]. Similar to other investigations [16], the average mesiodistal width of both sides of the dental arch was used in all of our statistical analyses. However, Salehi et al. [11] and Legovic et al. [17] only assessed the right and left quadrants, respectively.

In the linear regressions shown as Y = aX + b, the coefficient of determination (r^2^) indicates the correctness of estimating the equations to obtain Y (the sum of the mesiodistal width of canine and premolars) based on X (the mesiodistal width of the desired tooth or teeth, For example the mesiodistal width of four mandibular incisor teeth). This coefficient, sometimes expressed as a percentage, shows the proportion of the overall variability of Y that is determined by X in each Eqs. [11, 18].

Many studies, reported a significantly larger mean size of teeth in both jaws in men than in women [11, 16, 19]. Some studies such as Eshghi et al. [7] and Salehi et al. [11], conducted their investigations for the two sexes separately, and provided new regressions. In contrast, our findings were reported based on the combination of both sexes due to the nonequal number of male and female samples and the relatively small number of samples.

In the current study, the Tanaka-Johnson formula overestimated on average 0.86 (r^2^ = 0.458) in the maxilla and 0.82 (r^2^ = 2.436) in the mandible, which was statistically significant (*P* < 0.001). Similarly, Salehi et al. [11] and Tudeh-Zaim et al. [20] also reported the overestimation of this formula when studying the population of southern Iran. Other studies carried out on Jordanian [21], Turkish [19], Saudi [22], Qatari [23], and Senegalese [24] populations discovered the same result. The results of Jaroontham et al. [25] in the Thai population were close to the findings of the Tanaka-Johnson formula and it is possible to use it in that population. Some studies have attempted to modify the Tanaka-Johnson equation for their population. In fact, these studies presented new linear regression equations according to the relationship between the total mesiodistal width of the canine and succedaneous premolars with the total mesiodistal width of the mandibular incisors [7, 11, 20]. Differences in the findings might be explained by the variety of tooth sizes in different ethnicities.

Hashim et al. [23] presented that levels of 15%, 25%, and 35% of the Moyers table had more accurate results when two sexes were combined in the Qatari population. Al-khadra et al. [26] and Hashim et al. [22] studied the Saudi population and found that the 75% Moyers level was overestimated. Each of them obtained the best results from the 35% and 50% levels. Melgaco et al. [27] discovered underestimation of the 50% and 75% levels of the Moyers table in Brazilian people.

Moreover, Salehi et al. [11], found a significant difference in all levels of the Moyers table, which indicated the inaccuracy of this table in the population of southern Iran. Fatahi et al. [28] also found underestimation of all levels of the Moyers tables in Iranians and presented a suitable table for their studied population. The 75% level of Fattahi’s study was closest to the 85% level of Moyers (95% in the maxilla of men). They stated that using Moyers tables in space analysis for the Iranian population increases the possibility of lack of space during preventive orthodontic treatments, especially space supervision, and weakens the prognosis of the treatment. In our study, the values obtained from the Moyers table with 75% and 85% levels for the maxilla, and 75% and 65% for the mandible were the most similar. The 85% level was statistically and clinically similar in the maxilla. However, the 65% level had statistically better results, but clinically a small amount of overestimation was more favorable; thus, the 75% level was considered more appropriate. The similarity between these investigations might be explained by the fact that all of them worked on Iranian population.

Some studies did not consider it efficient to use only the total mesiodistal width of mandibular incisors in estimating the mesiodistal width of unerupted canines and premolars [20, 29], and as a result, several studies were conducted in different populations using other combinations of teeth to provide new regressions. Nourallah et al. [30] measured the correlation coefficient (r) between different groups of teeth. Some of these patients were excluded due to complicating factors such as the gingival covering of the distal first molar of the mandible, the late eruption of some teeth, and morphological obstacles, such as permanent lateral malformation of the maxilla. They presented a new regression equation based on the total mesiodistal width of the mandibular central incisor and maxillary first molar, which had a high correlation coefficient (r) and determination coefficient (r^2^). Tudeh-Zaim et al. [20] also presented a new regression. Similarly, we obtained new regressions based on these teeth as Y_x_=0.613X + 2.23 (*r* = 0.679) and Y_m_=0.618X + 1.6 (*r* = 0.679) for the upper and lower teeth, respectively. However, our obtained correlation coefficient was much higher than theirs.

Some investigations [14, 29] obtained new regression equations based on the total mesiodistal width of the maxillary central incisor and mandibular first molar. Additionally, we also examined this combination and obtained new regressions as Y_x_=0.423X + 5.031 (*r* = 0.630) and Y_m_=0.447X + 3.631 (*r* = 0.660) for the maxilla and mandible, respectively. Nevertheless, our correlation coefficient was higher than Ibrahim et al.’s [29] but lower than Prades et al.’s study [13].

On the other hand, Cattaneo et al. [31] found a new regression using 14 combinations of teeth (the maxillary lateral was excluded). The highest correlation coefficient was shown by the combination of mandibular incisors with the maxillary first molar. In addition, Melgaco et al. [32] and Shah et al. [33] both used the combination of incisors and mandibular first molars to create a new regression to estimate the mesiodistal width of mandibular canines and premolars.

In line with other studies, simple linear regressions (univariate) were used in the present investigation. Some studies chose multivariate linear regressions instead of simple ones [17, 34]. Multivariable equations have a higher correlation coefficient and accuracy; however, they are complicated and difficult to remember [11, 20, 32]. Multivariable equations are not suitable for clinical use and are only used for space analysis software [11].

Having a small sample size due to difficulty finding patients who met the inclusion criteria was the main limitation of the present investigation. Furthermore, various combinations of teeth should be tested in future studies considering the existing limitations in the mixed dentition period to obtain the appropriate regression for estimating the mesiodistal width of canines and premolars.

Suggesting novel linear regressions suitable for northern Iranian population was one of the strengths of this study, which has not been done on the same population before. Moreover, the current investigation was conducted on both sides of the dental arch which makes it more efficient. These findings can help orthodontics in estimating the required space more accurately and provide the patients the most convenient treatment plan.

## Conclusion

The present study discovered that the Tanaka-Johnson formula overestimates the space required for the growth of canine and premolars in northern Iran. The confidence levels of 75% of the Moyers table for the maxilla and 65% for the mandible had the best estimates statistically; however, the confidence levels of 85% and 75% for the maxilla and mandible showed better clinical results, respectively. Moreover, the linear regressions based on the total mesiodistal width of first molars and incisors in each jaw are as follows:

(I) The total mesiodistal width of the maxillary first molar and mandibular central incisor:
  *Maxilla*: *Y*_*x*_=*0.613X + 2.23*
  *Mandible*: *Y*_*m*_=*0.618X + 1.6*

(II) The total mesiodistal width of the mandibular first molar and maxillary central incisor:
  *Maxilla*: *Y*_*x*_=*0.423X + 5.021*
  *Mandible*: *Y*_*m*_=*0.447X + 3.361*

## Data Availability

The datasets used and/or analyzed during the current study available from the corresponding author on reasonable request.
